# P-1071. Renal Hyperfiltration Increases Mortality in patients with Multidrug-Resistant Gram-Negative Bacterial Pneumonia Treated with Standard-Dose Ceftazidime-Avibactam

**DOI:** 10.1093/ofid/ofaf695.1266

**Published:** 2026-01-11

**Authors:** Ming-Ying Ai, Wei-Lun Chang, Ming-Shyan Wang, Hong-An Chen

**Affiliations:** Far-Eastern Memorial Hospital, New Taipei, Taipei, Taiwan (Republic of China); Far-Eastern Memorial Hospital, New Taipei, Taipei, Taiwan (Republic of China); Far-Eastern Memorial Hospital, New Taipei, Taipei, Taiwan (Republic of China); Far-Eastern Memorial Hospital, New Taipei, Taipei, Taiwan (Republic of China)

## Abstract

**Background:**

Renal hyperfiltration may lead to subtherapeutic drug exposure in critically ill patients receiving standard doses of ceftazidime-avibactam (CAZ-AVI) for multidrug-resistant gram-negative bacterial (MDR-GNB) pneumonia. Previous studies have suggested that enhanced renal clearance results in reduced serum concentrations of both ceftazidime and avibactam, potentially compromising therapeutic efficacy. This study evaluates the impact of renal function on mortality in this patient population treat with standard dose of CAZ-AVI.

**Methods:**

This retrospective cohort study was conducted at the medical center in Taiwan and included patients treated with CAZ-AVI for MDR-GNB pneumonia between December 2019 and July 2024. Renal function was estimated using both the MDRD equation and the Cockcroft-Gault equation. The primary outcome was 15-day all-cause mortality, comparing patients with normal renal function to those with renal hyperfiltration (CrCl ≥130 mL/min). Secondary outcomes included the identification of risk factors for mortality using Cox proportional hazards regression. Kaplan-Meier survival analysis was performed to assess survival trends. Statistical analyses were conducted using SPSS version 19.

**Results:**

A total of 74 patients were included (median age: 69.4 years; 73% male). Renal hyperfiltration was identified as a significant risk factor for mortality in patients with MDR-GNB pneumonia receiving standard-dose CAZ-AVI (HR: 10.967, p< 0.001). Patients with CrCl ≥130 mL/min had significantly higher mortality rates when renal function was assessed using the MDRD equation (p< 0.001), likely due to suboptimal drug exposure. Similar trends were observed when renal function was evaluated using the Cockcroft-Gault equation (p=0.0461).

**Conclusion:**

Renal hyperfiltration (CrCl ≥130 mL/min) is associated with increased mortality in critically ill patients with MDR-GNB pneumonia treated with standard dose CAZ-AVI, likely due to inadequate drug exposure. Therefore, individualized dosing strategies based on renal function may be necessary to optimize drug exposure and improve clinical outcomes in this high-risk population.

**Disclosures:**

All Authors: No reported disclosures

